# Seasonal Profile and Five-Year Trend Analysis of Malaria Prevalence in Maygaba Health Center, Welkait District, Northwest Ethiopia

**DOI:** 10.1155/2021/6727843

**Published:** 2021-09-10

**Authors:** Fitsum Tigu, Tsegay Gebremaryam, Asnake Desalegn

**Affiliations:** ^1^Department of Microbial, Cellular and Molecular Biology, Division of Microbiology, College of Natural Sciences, Addis Ababa University, Addis Ababa, Ethiopia; ^2^Department of Zoological Sciences, College of Natural Sciences, Addis Ababa University, Addis Ababa, Ethiopia

## Abstract

**Background:**

Malaria is a serious public health problem of most developing countries, including Ethiopia. The burden of malaria is severely affecting the economy and lives of people, particularly among the productive ages of rural society. Thus, this study was targeted to analyze the past five-year retrospective malaria data among the rural setting of Maygaba town, Welkait district, northwest Ethiopia.

**Methods:**

The study was done on 36,219 outpatients attending for malaria diagnosis during January 2015 to 2019. Data was extracted from the outpatient medical database. Chi-square (*χ*^2^) test and binary logistic regression model were used to analyze the retrospective data. Statistical significance was defined at *p* < 0.05.

**Results:**

Of 36,219 outpatients examined, 7,309 (20.2%) malaria-positive cases were reported during 2015-2019. There was a fluctuating trend in the number of malaria-suspected and -confirmed cases in each year. Male slide-confirmed (61.4%, *N* = 4,485) were significantly higher than females (38.6%, *N* = 2,824) (*p* < 005). *Plasmodium falciparum* and *Plasmodium vivax* were the dominant parasites detected, which accounted for 66.1%; *N* = 4832, 33.9%; *N* = 2477, respectively. Despite the seasonal abundance of malaria cases, the highest prevalence was recorded in autumn (September to November) in the study area. Binary logistic regression analysis revealed that statistically significant associations were observed between sexes, interseasons, mean seasonal rainfall, and mean seasonal temperature with the prevalence of *P. vivax*. However, *P. falciparum* has shown a significant association with interseasons and mean seasonal temperature.

**Conclusions:**

Although the overall prevalence of malaria was continually declined from 2015-2019, malaria remains the major public health problem in the study area. The severe species of *P. falciparum* was found to be the dominant parasite reported in the study area. A collaborative action between the national malaria control program and its partners towards the transmission, prevention, and control of the two deadly species is highly recommended.

## 1. Introduction

Malaria is one of the major tropical diseases that adversely affect the public health and the economic development of many developing countries [1]. There are five known species of *Plasmodium* (*P. falciparum*, *P. vivax*, *P. malariae*, *P. ovale*, and *P. knowlesi*) which are able to cause malaria in humans through the bite of female anopheles mosquitoes [2]. Among which, *P. falciparum* and *P. vivax* are the most deadly parasites that cause severe morbidity and mortality in most malarious regions of the world including Ethiopia [2, 3].

Malaria causes serious systemic complications in humans, including infectious in the nervous, respiratory, renal, and hematopoietic systems [4]. Particularly, the severity of *P. falciparum* malaria is much more complex than any other known species. The clinical complications include, but not limited to impaired consciousness, acidosis, hypoglycaemia, severe anaemia, renal impairment, jaundice, pulmonary oedema, significant bleeding, shock, and hyperparasitaemia [5]. Delayed treatment and lack of immediate supportive care may result in death.

Looking at the world malaria cases from 2010 to 2018, there was a marked decline with the exception of WHO African region. This region still carries the highest share of global malaria burden [6]. The root cause of malaria in this region is always associated with lower socioeconomic status, food insecurity, poor housing, and lack of medical care [7]. Despite the fact that malaria is associated with poverty, it is also strongly associated with environmental conditions [8]. The presence of conducive mosquito breeding sites and suitable temperature for the parasite as well as for the vector developments was frequently reported in the tropical regions of Africa [9].

In Ethiopia, the distribution and transmission of malaria are largely unstable and seasonal and dependent on altitude and climate. In addition, the majority of the people are exposed to malaria due to the presence favorable topography and climate for malaria transmission [9–11]. Ethiopia has recorded an estimated 2,927,266 new malaria cases and 4,782 deaths with a crude death rate of 4.7 per 100,000 in 2016 [12].

However, in recent decades, Ethiopia has achieved notable progress in fighting against malaria and significantly reduced malaria-related morbidity and mortality. This was achieved through coordinated efforts of the National Malaria Control and Elimination Program (NMCEP) and its partners [13]. It was tackled by intensive use of insecticide residual spray (IRS), long-lasting insecticide-treated mosquito nets (LLNs), chemotherapies, improved diagnosis, and case management and provision of quality laboratory facilities to the patients [14].

The feasibility of malaria elimination in Ethiopia and making a malaria-free Ethiopia requires concurrent investments in the high burden areas in the west, and data-informed decision-making is crucial to these efforts [13, 14]. Therefore, the aim of this study was to assess the prevalence and trend of malaria among the rural setting of Maygaba town, Welkait district, northwest Ethiopia from 2015 to 2019. Additionally, this study will give evidence-based information about the current status of malaria to all stakeholders and provide useful data for decision makers, policy makers, and malaria prevention and control strategists for the design and implementation of possible solutions.

## 2. Methods

### 2.1. Study Design and Setting

The study was designed to analyze a five-year (2015-2019) retrospective data extracted from Maygaba Health Center (MHC). The study was conducted in Maygaba town of Welkait district, northwest Ethiopia. The town is located about 929 km from the capital city, Addis Ababa, and 273 km from Gondar city. It has one health center and six health posts. According to the Myagaba town communication office, the total population of the study area is about 30,974, of which 15,642 are males and 15,332 are females. The altitude of the study area ranges between 677 and 2,755 meters above sea levels. The mean annual temperature is 22.5°C with minimum and maximum temperature ranges of 15 and 30°C, respectively. The town and its surrounding rural areas had a total of six villages, one urban and five rural villages with 7,039 households. Each village had an average family size of 4.4 persons per household.

### 2.2. Data Collection

For the past five years (2015-2019), malaria health examination record data were collected from MHC using the format developed by the principal investigator. The format includes malaria diagnosis results (negative or positive), types of infecting *Plasmodium* species (for positives), year of diagnoses, and sociodemographic data. However, due to the incompleteness of the medical record sheets, the sociodemographic data were excluded from the analysis except the sex. The malaria trends in the study area were analyzed by appropriate software.

### 2.3. Data Analysis

Wholeness and consistency of the data were checked twice and entered into SPSS version 20 software (SPSS Inc., Chicago, IL, USA) for statistical analysis. Descriptive statistics (frequencies and percentages) were used to describe the malaria cases with respect to months, years, sex, and *Plasmodia* species. Chi-square (*χ*^2^) test and binary logistic regression model were applied to analyze the association of the different variables with confirmed malaria cases. Statistical significance was defined at *p* values < 0.05.

## 3. Results

### 3.1. Trends of Malaria Cases in MHC

**Figure 1:**
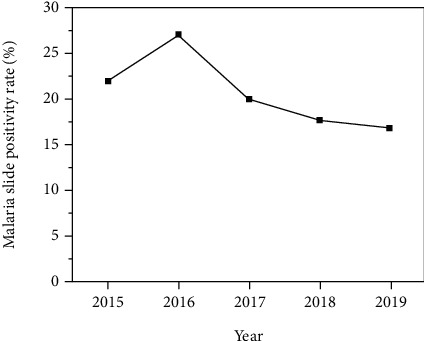
Trends of malaria prevalence at MHC, northwest Ethiopia, from 2015-2019.

During January 2015 to December 2019, about 36,219 patients have been diagnosed for malaria and out of which 20.2% (*N* = 7,309) were slide-positive. On average, 7,244 malaria-suspected and 1,462 malaria-confirmed cases were tested by MHC every year. Variation in the number of malaria-suspected and -confirmed cases in each year was observed. Despite the variation, the average monthly malaria prevalence was 1.7%. Generally, the overall prevalence of malaria showed a declining trend during 2015 to 2019, except a slight increase recorded in 2016 (Figure 1 and Table 1).

### 3.2. Distribution of Confirmed Malaria Cases by Sex

Regarding the distribution of sex, from the total tested cases, slightly over half (54.7%, *N* = 19,797) of patients were males and (45.3%, *N* = 16,422) were females. While from the total slide-confirmed cases, the majority (61.4%, *N* = 4,485) of them were males and the rest (38.6%, *N* = 2,824) were females.

Looking at the overall male to female ratio of tested and slide-confirmed cases, mostly males were affected more frequently than females with a ratio of 1.2 : 1 and 1 : 0.6, respectively. This difference was statistically significant (*χ*^2^ = 3.923, *p* < 0.05). Consequently each year, a higher number of malaria-positive males were observed than the malaria-positive females; however, yearly difference in the number of cases was not statistically significant (*p* > 0.05). Comparing the annual overall prevalence of malaria under each sex category, males showed a higher (22.7%) prevalence than females (17.2%) (Table 2).

### 3.3. Seasonal Profile of Malaria Prevalence

**Figure 2:**
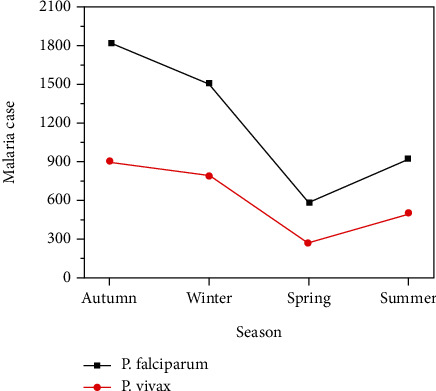
Seasonal prevalence of malaria by *Plasmodium* types at MHC, northwest Ethiopia, from 2015-2019.

The total number of malaria examined over malaria positive cases showed great variation between seasons. With respect to the number of cases, total cases exhibited the following order: autumn > winter > summer > spring (Table 3 and Figure 2). Majority of suspected as well as infected cases were observed soon after the main rainy season (September to November). A total of 33.3% of malaria negative and 37.5% malaria positive cases were reported in this season (Figure 2 and Table 3). While the smallest number of malaria-suspected (17.8%) and -infected (11.7%) cases were reported during the small rainy season (March-May). Almost the same malaria-suspected cases (25.5 and 23.8%) were observed in dry and heavy rainy seasons, respectively (Figure 2 and Table 3). A significantly higher number of slide-confirmed malaria cases (37.5%) were observed during autumn soon after the heavy rainy season (*p* < 0.01) compared to dry and small rainy seasons.

Generally, pairwise comparisons indicate that there are significant interseasonal variations (*p* < 0.001) except between autumn and summer (*p* = 0.376). Since the actual season of malaria in the study area was not clearly defined, it is not possible to calculate the prevalence of malaria for each season. Nevertheless, this imperative result tells us that malaria was observed throughout all the seasons.

### 3.4. Plasmodium Species Distribution by Years and Seasons

**Figure 3:**
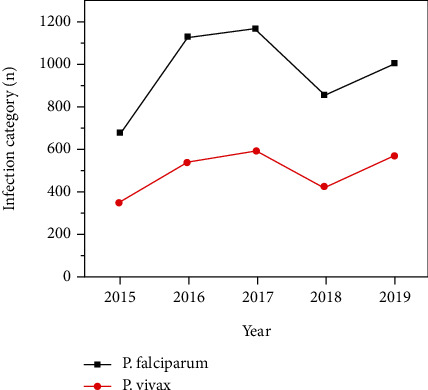
Side positive cases by infection category at MHC, northwest Ethiopia, from 2015-2019.

In terms of infectious category, *P. falciparum* was the major (66.1%) contributor to malaria infection in the study area, while *P. vivax* accounted only (33.9%) of infection (Table 3). The difference was statistically significant (*χ*^2^ = 758.8, *p* < 0.001). Coinfection of *P. falciparum* and *P. vivax* was not reported at all during the 2015-2019 retrospective study. The overall trend of both infections showed variation between years and seasons. Apparently, *P. falciparum* was the dominant infection that occurred consistently over 66% in all study years and seasons (Figure 3 and Table 3). So, it is not surprising that *P. falciparum* was the most prevalent infection than *P. vivax* in the past five years at the study site.

Despite dominance and prevalence, both infections followed similar patterns towards seasonality (Figure 2 and Table 3). About 37.8, 31.2, and 19.1 of *P. falciparum* infection were registered in September to November, December to February, and June to August, respectively. Similarly, about 36.9, 31.8, and 20.3 of *P. vivax* cases were also recorded in September to November, December to February, and June to August, respectively (Figure 2 and Table 3). Although a lower number of *P. falciparum* (12%) as well as *P. vivax* (11%) cases was reported in the small rainy season than any other seasons, however, statistically significant difference was not noticed (*χ*^2^ = 5.67, *p* = 0.234).

### 3.5. Correlation between Malaria Type and Other Predictor Variables

The relationship between malaria type and other predictor variables (sex, season, rainfall, and temperature) was analyzed by a binary logistic regression model as indicated in Table 4. It showed that the inert-seasons and mean seasonal temperature have statistically significant (*p* < 0.001) associated with the prevalence of *P. falciparum.* Other independent factors such as sex and mean seasonal rainfall have no significance associated with it. However, statistically significant (*p* < 0.001) associations were observed between sexes, interseasons, mean seasonal rainfall, and mean seasonal temperature with the prevalence of *P. vivax*. In addition to this, significantly higher numbers of *P. vivax* cases were observed during autumn and winter seasons compared to that of summer and spring seasons. Also, significant differences were observed between males and females, where males were more affected by *P. vivax* compared to females (AOR = 0.754, 95%CI = 0.681 − 0.835, *p* < 0.001).

## 4. Discussion

Malaria is the major public health concern in Ethiopia and causes significant morbidities and mortalities to the people. Particularly, the rural societies are extremely affected by this vector borne disease. Thus, the purpose of this trend analysis was to investigate the current status and impact of malaria on the rural settings across the years and seasons from 2015 to 2019. Ultimately, the findings of this research will pave the way to the creation of malaria-free-nation in Ethiopia.

The five-year retrospective data analysis revealed that the overall prevalence of malaria was showing a declining trend except a slight increase observed in the year of 2016, and this was due to the occurrence of unseasonal rainfall in the study area as the information obtained from the MHC indicates but significant association was not observed with yearly mean rainfall data (data not shown). This might be because the rainfall data used for analysis was obtained from nearby city (Gondar), which is far by 273 km from the study site due to the lack of meteorological data in the exact study site.

A total of 7309 (20.2%) of slide-confirmed malaria cases were recorded during the five-year study period. This finding was comparable with the study conducted in the East Wollega, 20.0% [15] and the Northwest Gondar, 21.8% of Ethiopia [16]. However, the presence of parasitemia in this study was quite lower than similar studies conducted in various regions of Ethiopia ranged from 32.6 to 41.5% [17–20] and higher than the findings in the North Shoa, South Wello, and West Gojam Amhara region and North West Tigray of Ethiopia it ranged from 5.0 to 16.3% [21–26]. The possible reasons for the differences may be due to variations of the study settings, time of the investigation, LLN coverages, and the knowledge levels of the societies and interventions of malaria prevention and control measures.

The health record data of this study indicated that a significant number of male malaria-confirmed cases were recorded in the MHC compared to the female, suggesting that males were extremely infected by malaria in the study area. This finding was comparable with a similar study reported in another malaria-endemic area of Ethiopia [27]. This is because males' had a greater occupational risk of getting the disease than women. Males, mainly 18-40 years old, are usually engaged in outdoor activity such as farming particularly, irrigation activity was mostly done during evening up to night in the study area. However, logistic regression analysis conducted between malaria type and sexes indicated that only *P. vivax* has a significant correlation (AOR = 0.754; 95%CI = 0.681 − 0.835; *p* < 0.001) with sexes.

Malaria transmission was recorded throughout the seasons in the study area, and the interseasonal variations were significant (*p* < 0.001). The substantial number of malaria-infected cases was reported shortly after the main rainy season. This is because the existence of cold and cloudy weather conditions during this season creates a conducive environment for breeding of mosquitoes [28]. Furthermore, most cultivated crops including maize release pollen grains following the rainy season may serve as a food source for mosquito larvae to complete its life cycle.

In this study, *P. falciparum* was predominantly found in the study area and became a major (66.1%) contributor of morbidity and mortality followed by *P. vivax*. This was in agreement with the national report [29, 30] as well as reports from most other regions of Ethiopia [3, 31, 32], but in contrast to the closest region of Africa, Eastern Sudan [33]. This might be due to the fact that the pathogenesis of *P. falciparum* is stronger than the *P. vivax* [34, 35].

The prevalence of malaria in Ethiopia largely depends on seasons [11], and the highest transmission of both parasites (*P. falciparum* and *P. vivax*) is also recorded biannually following the two rainy seasons (September to November and March to May) [27]. The coincidence of malaria transmission patterns with the peak agricultural activities significantly affects the economy of the country [32]. However, in this study, the lowest prevalence was reported in the small rainy season (March to May). This finding further strengthens the variabilities of malaria transmission in Ethiopia [36, 37].

Furthermore, binary logistic regression analysis showed that the parasite prevalence of *P. falciparum* and *P. vivax* has strongly correlated with meteorological data such as mean seasonal temperature and mean seasonal rainfall except the latter was not significantly associated with the prevalence of *P. falciparum*. This might be due to the fact that temperature, rainfall, and relative humidity are the major climatic factors that have an impact on malaria transmission [15]. However, the absence of a positive correlation of *P. falciparum* with rainfall in this study may be due to variable effects of the rainfall in hot areas and cold areas. The effect of rainfall in hot areas towards the prevalence of *P. falciparum* was relatively weaker than the cold areas as reported [38]. This study was in agreement with the ten-year retrospective study conducted in Western Ethiopia [15].

In the epidemiological point of view, rainfall has played an important role in development and reproduction of adult mosquitoes [39]; however, the impact of rainfall on the transmission of malaria is very complicated since the effect is dependent on various situations such as geographical region and specific to breeding habitats of mosquitoes. A study conducted by McMichael [39] indicated that moderate rains may have beneficial to mosquito breeding while heavy rains may clear out the breeding sites as well as the developing mosquito larvae.

Finally, it is difficult to draw better and reliable conclusions from five years of health recorded data, and analyzing as much recorded data as possible may provide more qualified conclusions. However, due to poor recordkeeping at MHC and lack of malaria-related follow-up data, for instance, age, morbidity, and mortality data were missing and limited our study. Furthermore, the meteorological data used in this study for binary regression analysis was obtained from nearby city, Gondar, due to a lack of data in the exact study site.

## 5. Conclusions

Although the overall prevalence of malaria was declined during the study period of 2015 to 2019, malaria remains the major public health problem in the study area. The severe species of *P. falciparum* was found to be the dominant parasite reported during the study periods. Autumn (September to November) was identified as the highest malaria prevalence season, and males are more affected than females in the study area. A collaborative action between the national malaria control program and its partners towards the transmission, prevention, and control of the two deadly species is highly recommended.

## Figures and Tables

**Table 1:** Annual trends of total malaria cases in MHC, from 2015-2019.

Year	Blood films examined	Slide-confirmed malaria cases (%)
2015	4642	1021 (22.0)
2016	6153	1667 (27.1)
2017	8850	1764 (19.9)
2018	7255	1281 (17.7)
2019	9319	1576 (16.9)
Total	36219	7309 (20.2)

**Table 2:** Distribution of confirmed malaria cases by sex at MHC, from 2015-2019.

Sex	Total cases	Slide positive (*n*, %)	*P. falciparum* (*n*, %)	*P. vivax* (*n*, %)	*χ* ^2^	*p* value
Male	19797	4485 (61.4)	3010 (67.1)	1475 (32.9)	3.923	0.04
Female	16422	2824 (38.6)	1822 (64.5)	1002 (35.5)		
Total	36219	7309	4832 (66.1)	2477 (33.9)		

**Table 3:** Seasonal profile and prevalence of malaria at MHC during 2015-2019.

Season	Total cases	Total positive (%)	Total negative (%)	*P. falciparum* (%)	*P. vivax* (%)
Summer (Jun. to Aug.)	8615	1424 (19.5)	7191 (24.9)	921 (19.1)	503 (20.3)
Autumn (Sep. to Nov.)	12371	2739 (37.5)	9632 (33.3)	1825 (37.8)	914 (36.9)
Winter (Dec. to Feb.)	9224	2293 (31.4)	6931 (24.0)	1506 (31.2)	787 (31.8)
Spring (Mar. to May)	6009	853 (11.7)	5156 (17.8)	580 (12.0)	273 (11.0)
Total	36219	7309	28910	4832 (66.1)	2477 (33.9)

**Table:** (a) With *Plasmodium falciparum*

Variable	AOR	95% CI	*p* value
Gender			
Male	1		
Female	1.074	0.72-1.188	0.163
Season			
Summer (Jun. to Aug.)	1		
Autumn (Sep. to Nov.)	2.557	1.403-2.770	<0.001
Winter (Dec. to Feb.)	1.528	1.422-1.661	<0.001
Spring (Mar. to May)	0.581	0.461-0.731	<0.001
Mean seasonal rainfall	0.980	0.936-1.025	0.379
Mean seasonal temperature	0.788	0.744-0.836	<0.001

**Table:** (b) With *Plasmodium vivax*

Variable	AOR	95% CI	*p* value
Gender			
Male	1		
Female	0.754	0.681-0.835	<0.001
Season			
Summer (Jun. to Aug.)	1		
Autumn (Sep. to Nov.)	2.129	1.698-2.669	<0.001
Winter (Dec. to Feb.)	1.966	1.556-2.484	<0.001
Spring (Mar. to May)	0.731	0.651-0.995	<0.001
Mean seasonal rainfall	1.051	1.003-1.101	0.038
Mean seasonal temperature	1.297	1.229-1.369	<0.001

## Data Availability

All relevant data are within the paper.

## Abbreviations

MHC: Maygaba Health Center
LLNs: Long-lasting insecticide-treated mosquito nets
CNCS-IRB: College of Natural and Computational Sciences, Institutional Review Board
NMAE: The National Meteorological Agency of Ethiopia.
